# Angular- and Polarization-insensitive Ultrathin Double-layered Metamaterial Absorber for Ultra-wideband Application

**DOI:** 10.1038/s41598-018-28041-5

**Published:** 2018-06-25

**Authors:** Li Li Cong, Xiang Yu Cao, Tao Song, Jun Gao, Jun Xiang Lan

**Affiliations:** 1 Information and Navigation College, Air Force Engineering University, Xi’an, 710077 China; 2 Air Defense and Missile College, Air Force Engineering University, Xi’an, 710077 China

## Abstract

We proposed an ultra-thin polarization-insensitive metamaterial absorber (MMA) for ultra-wideband and wide incident angle operation. The MMA is composed of double-layer symmetric split rings (SSRs) connected with two orthogonally-arranged bars and the ground metallic plane separated by two identical substrates. Multiple metallic layers and scalabilities are employed to provide broadband absorptivity based on the cooperated mechanisms of the Ohmic loss and the Fabry-Perot interference. To further broaden the absorption bandwidth, four lumped resistors are loaded with the SSRs on the top metallic layer. By this means, an ultra-wideband absorbance is achieved nearly in 4~22 GHz, two gentle slope belts with absorptivity over 60% and 80% in 4~12 GHz and 12~22 GHz, respectively. The whole structure is with an ultrathin thickness of 2.4 mm, which is 0.032λ_low_ and 0.176λ_high_ corresponds to the lowest and highest absorption frequency separately. Meanwhile, the symmetric structure enables the MMA of satisfactory stability for polarization and wide incident angles. Numerical and experimental results prove the capability of the proposed MMA for ultra-wideband absorbance.

## Introduction

Electromagnetic (EM) metamaterials (MTMs), consisting of a periodic array of sub-wavelength resonators^1–4^, have been extensively applied for EM compatibility and military applications. The investigation of MTM into EM absorbers has sparked increasingly growing concern^5–7^ since Landy *et al*. first introduced the concept of perfect metamaterial absorber (MMA) in 2008^8^. In 2012, C. M. Watts *et al*.^9^ give an overview of the progress of MMA in the EM field from microwave to optical range. By demonstrating MMA design versatilities and related applications, the performance flexibility and underlying operation mechanism are discussed. Coincidently, in 2015 Y. Ra’di *et al*.^10^ concentrate on giving a general overview on the fundamental operation theory of thin MMA. Design equations and related realizations are provided for each identified class of thin MMA. In recent years, with rapid advancement of processing technology, some new type MMAs provide an alternative route to perform perfect absorption, including nonlinear saturable MMA^11^, 3-D totally-metal MMA without dielectric^12^, coherent perfect absorber in purely imaginary MTMs^13,14^, etc.

In contrast with bulky conventional absorbers^15,16^, MTM-based absorbers have such advantages as ultrathin thickness, light configuration, good conformal shape and easy fabrication^17–20^. Such advantages of MMAs render them potential candidates for bolometers, anechoic chamber and cloaking. Moreover, the most promising application of MMAs is EM stealth. By optimizing the geometrical parameters of a single unit, the effective EM properties, viz. permittivity and permeability, can be modified in such a way that for an incidence the input impedance of the unit cell becomes equal to that of free space, which results in nearly unity absorption^8,21^. However, some undesired performances, such as narrow bandwidth for a given thickness^8,22,23^, polarization-sensitivity and narrow incident angle^24–26^, restrict wide application of MMA. Especially, narrow bandwidth resulting from the resonance and high quality factor has been the main obstacle for wide application of MMAs. Many methods have been proposed to broaden the absorption bandwidth. One is to adopt passive frequency selective surface (FSS) loaded with lumped resistors^27,28^ and capacitors^29^, or active FSS loaded with pin diodes^30^, varactor diodes^31^ or non-Foster loads^32^. The second is to utilize orthogonally arranged multi-scale unite cells^33,34^ or multiple vertically stacked metallic layers^22,35,36^. The last but not least is to use high loss substrate or resistive sheet or magnetic composite sheets^18,37,38^. Nevertheless, the absorption bandwidth enhancement is still limited to some extent. Several other works have devoted to improve MMA performances in terms of angular stability^39^ and polarization insensitivity^40^. Broadband MMA collaborated with angular-stability and polarization-insensitivity capabilities are barely studied. A synthesis approach to design an ultra-thin MMA integrated with wide incident angle, polarization insensitivity and broadband application is urgently needed. This paper is proposed to address this problem.

In this paper, a double-layered polarization-independent wide-angle incidence MMA capable of ultra-wideband operation is proposed. The basic unit cell is a 2 × 2 array of four-fold symmetric split rings (SSRs) connected with two orthogonally-arranged bars in multiple scales. Multilayer and multi-scalability are combined together to realize a broadband and high efficient absorptivity. To further improve the absorption performance, four lumped resistors are added to the gaps between the SSRs on the top metallic surface. In this way, an ultra-wideband absorbance with two gentle slope belts absorptivity over 60% and 80% are respectively achieved in 4~12 GHz and 12~22 GHz through synergetic effects of the Ohmic loss and Fabry-Perot interference. Besides, the four-fold symmetric structure makes the proposed MMA insensitive to polarization and incident angles, which provides a good candidate for EM stealth application.

## Results

### Design and analysis of the MMA

**Figure 1:**
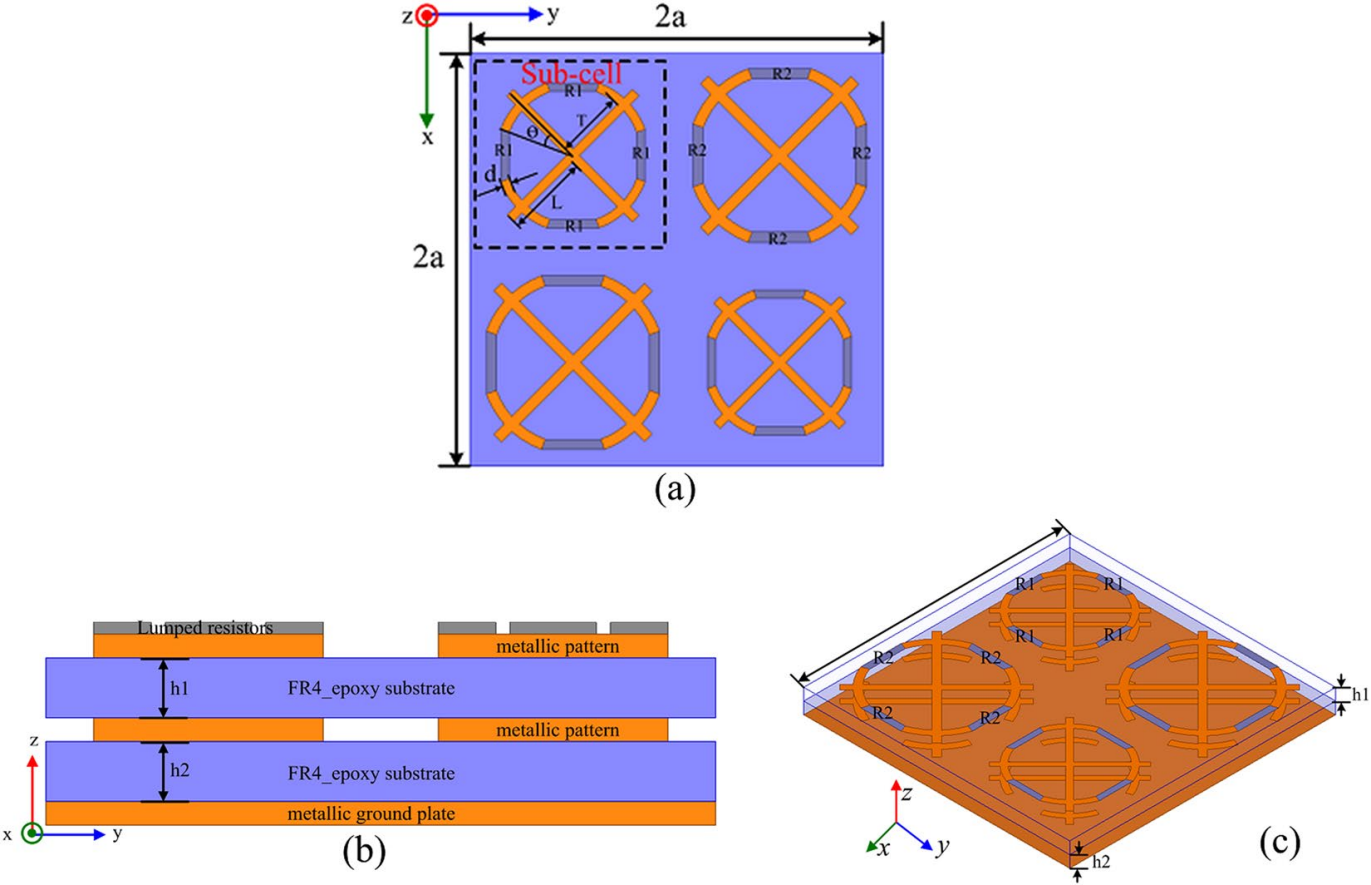
Schematic structure of the proposed MMA. (**a**) Top view, (**b**) side view and (**c**) perspective view.

The basic unite cell of the proposed MMA consists of two stacked substrates with same metallic patterns imprinted on top surface of them, while the whole structure is backed by a continuous metallic surface. Either of the metallic patterns comprises of a sub-array of two kind diagonally-arranged sub-cells. The sub-cell is made up of SSRs connected with two orthogonally-arranged bars. To expand the absorption bandwidth in the largest frequency band, four lumped resistors are mounted between gaps of the SSR on the top metallic surface, as shown in Fig. 1(a). It is clearly shown that excluding the lumped resistors the two kind sub-cells are of the same shape but different scalabilities, which could facilitate the design and fabrication process. An epoxy FR4 substrate with a dielectric constant of 4.4 and a loss tangent of 0.02 has been utilized as the dielectric layer. Both the substrates are with a thickness of 1.2 mm, contributing to a total thickness of 0.032λ_low_ and 0.176λ_high_ in respect to the lowest and highest absorption frequency. The three metallic layers are modeled as lossy copper with electric conductivity of σ_*copper*_ = 5.8 × 10^7^ S/m and a thickness of 0.035 mm. The side and perspective views of the basic unite cell are depicted in Fig. 1(b,c) respectively. The finally optimized parameters of the single sub-cell are listed in Table 1.

**Table 1:** Optimized dimensions of single sub-cell.

S. no.	Parameter	Dimension
1	a	14 mm
2	L	5.70 mm
3	T	4.60 mm
4	d	0.60 mm
5	h1	1.2 mm
6	h2	1.2 mm
7	θ	25 deg
8	R1	240 ohm
9	R2	240 ohm

As known to all, the efficiency of an absorber can be characterized as *A*(*ω*) = 1 − *R*(*ω*) − *T*(*ω*), where *A*(*ω*), *R*(*ω*), *T*(*ω*) stand for the absorptivity, reflectivity and transmission as functions of frequency *ω*, respectively. Obviously, it is desirable to minimize the reflection and transmission simultaneously to realize a perfect absorber with high efficiency. The reflection and transmission can be derived from *S*-parameters, where *R*(*ω*) equals to |*S*_11_(*ω*)|^2^, *T*(*ω*) equals to |*S*_21_(*ω*)|^2^. On one hand, the transmission is zero attributed to the continuous metal plane in the proposed design. On the other hand, *R*(*ω*) = (*Z*_*eff*_ − 1)/(*Z*_*eff*_ + 1), from which a conclusion can be drawn that the reflectivity *R*(*ω*) could be minimized if the total impedance of the structure matches that of free-space. Then the absorption can be rewritten as *A*(*ω*) = 1 − *R*(*ω*) = 1 − |*S*_11_(*ω*)|^2^. To better understand the absorption mechanism of our design, the effective impedance of the structure can be extracted from *S*-parameter given as *Z*_*eff*_(*ω*) = [*μ*_*eff*_(*ω*)/*ε*_*eff*_(*ω*)]^1/2^ = [((1 + *S*_11_)^2^ − *S*_21_^2^)/((1 − *S*_11_)^2^ − *S*_21_^2^)]^1/2 ^^41^. And then to be simplified as *Z*_*eff*_(*ω*) = [(1 + *S*_11_)^2^/(1 − *S*_11_)^2^]^1/2^. Besides, the completely symmetric structure along *x*- and *y*-axis makes the absorptivity insensitive to arbitrary polarizations of incident waves.

Numerical simulations were carried out using commercial simulation software Ansys HFSS to investigate the EM properties of the MMA. Floquet port excitations and master/slave boundaries are performed to simulate the infinite 2-D periodic boundary condition. To achieve the absorption characteristic in the largest frequency band, three main factors have been taken into account in the finally proposed design. One is the number of metallic layers, another is the multi-scalability, and the last is the load of lumped resistors. The effects of each factor on absorption performance are investigated in the following section. It is worth mentioning that there are some other factors affecting the absorption performance, such as arc angles of the SSRs, which is beyond the scope of this paper.

### Multiple metallic layers effect

**Figure 2:**
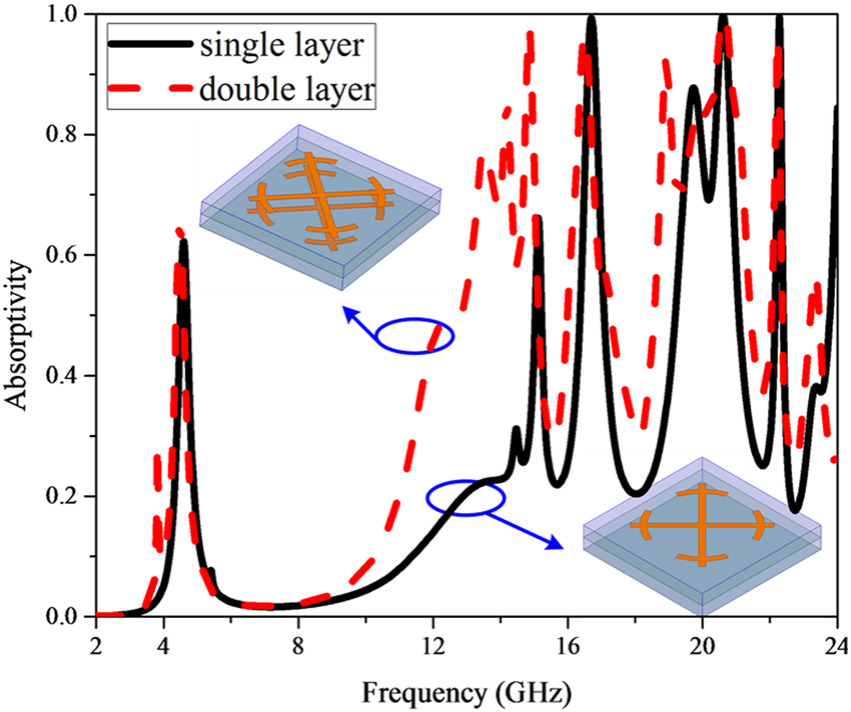
Absorption response versus frequency under normal incidence for single metallic layer and double metallic layers.

**Figure 3:**
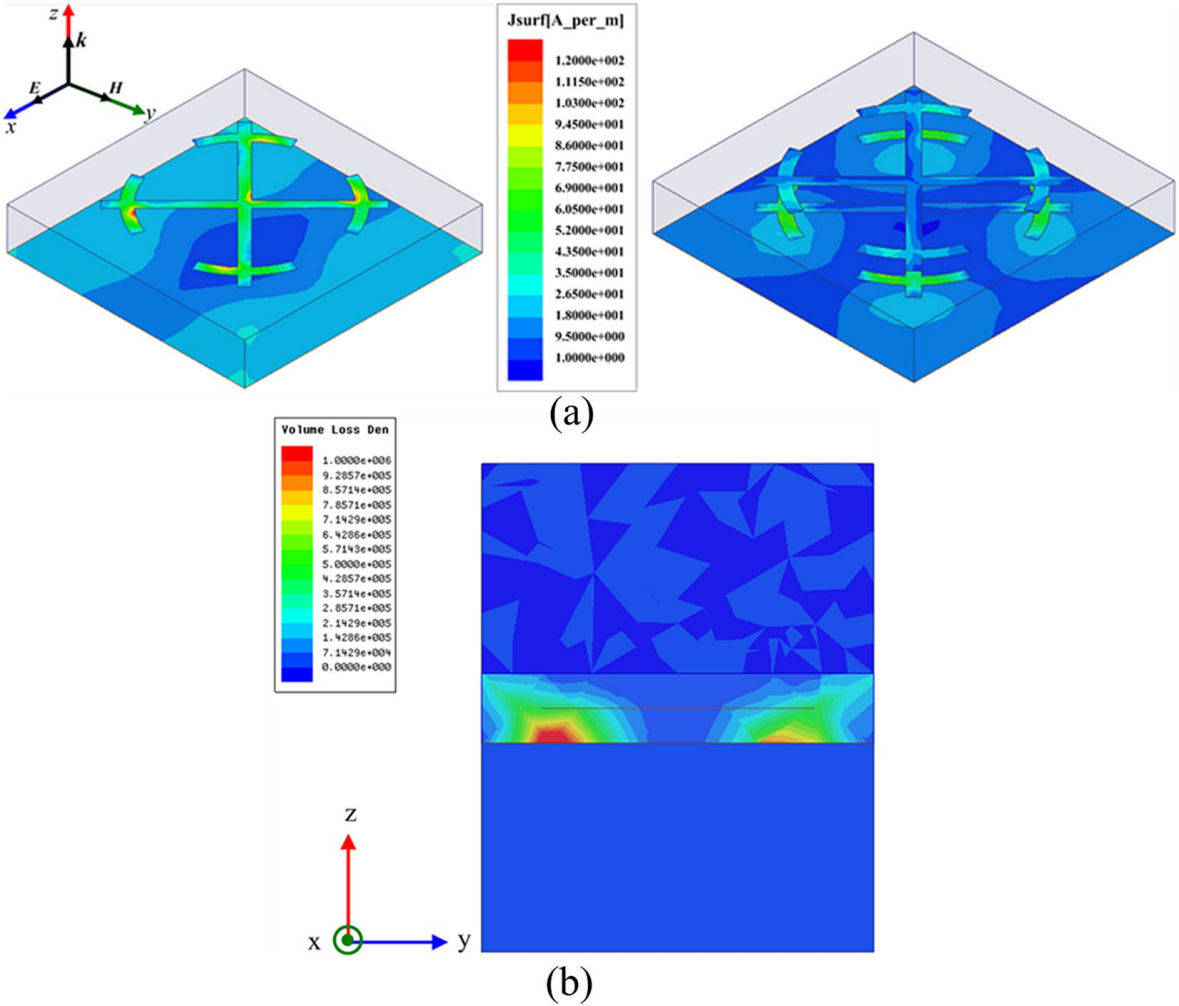
(**a**) Surface current distributions for single and double metallic layers excited by normal incidence at 13.5 GHz, and (**b**) Transverse view of the dielectric (volume) loss in double layered MMA at 13.5 GHz.

**Figure 4:**
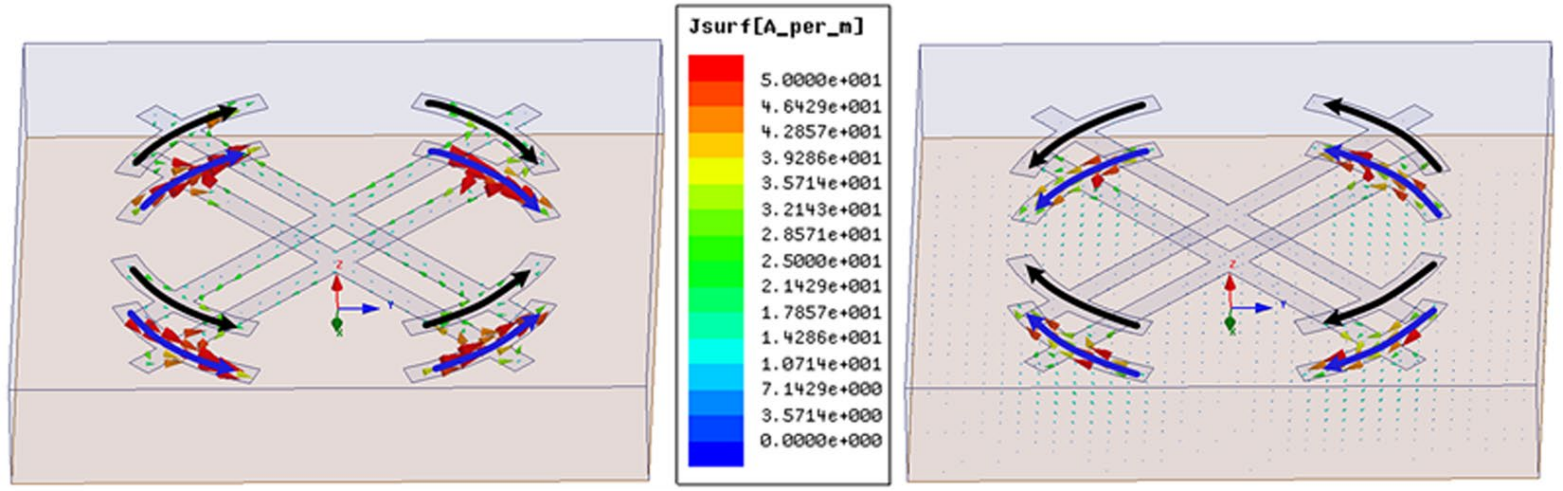
Current distributions across metallic surface of MMA with double metallic layers at 13.48 GHz and 14.2 GHz, respectively.

The simulated frequency response of sub-cell without resistors for normally incident EM wave is demonstrated in Fig. 2. For comparison, the simulated result of sub-cell with exactly same substrate and only the top metallic pattern has also been given. Apparently, compared with single metallic layer, the absorption performance has been improved as a whole, especially from 12 GHz to 15 GHz. There are ten absorption peaks valuing 0.29, 0.66, 0.78, 0.86, 0.98, 0.97, 0.92, 0.85, 0.999, 0.96, 0.57 located at 3.82 GHz, 4.48 GHz, 13.48 GHz, 14.18 GHz, 14.86 GHz, 16.50 GHz, 18.92 GHz, 19.92 GHz, 20.64 GHz, 22.24 GHz, 23.34 GHz, respectively. To better understand the absorption difference between these two configurations, the surface current distributions at 13.5 GHz are depicted in Fig. 3 (surface current distributions at 3.82 GHz are discussed in Supplementary Materials). Apparently, for single metallic layer, the surface current excited by 13.5 GHz EM wave mainly gathers on the four split rings and the central part of the bars, while resonant surface current mainly concentrates on the four split rings of the middle layer and relative position on the backing lattice for double metallic layers. From the transverse view of the volume loss density in the dielectric substrate, we can clearly see that the dielectric losses occur strongly in between the middle metallic layer and the backing ground, while with weakly in between the top and middle metallic layers. This phenomenon indicates that the Fabry-Perot interference^42^ forms in the double layered MMA. Multi-reflection occurs in the Fabry-Perot resonance cavity formed by the three metallic layers and the dielectric, thus resulting in high absorptivity. It is worth noting that when double metallic layers introduced in MMA, two adjacent splitting absorption peaks occur at 13.48 GHz and 14.2 GHz, respectively. We can conclude from the current distributions in Fig. 4 that the excited currents correspond to symmetric and anti-symmetric modes^43,44^, which leads to the resonance splitting. Meanwhile, the different efficiency of coupling between the modes and incident plane wave result in absorption peaks with different levels.

### Multiple scalabilities effect

**Figure 5:**
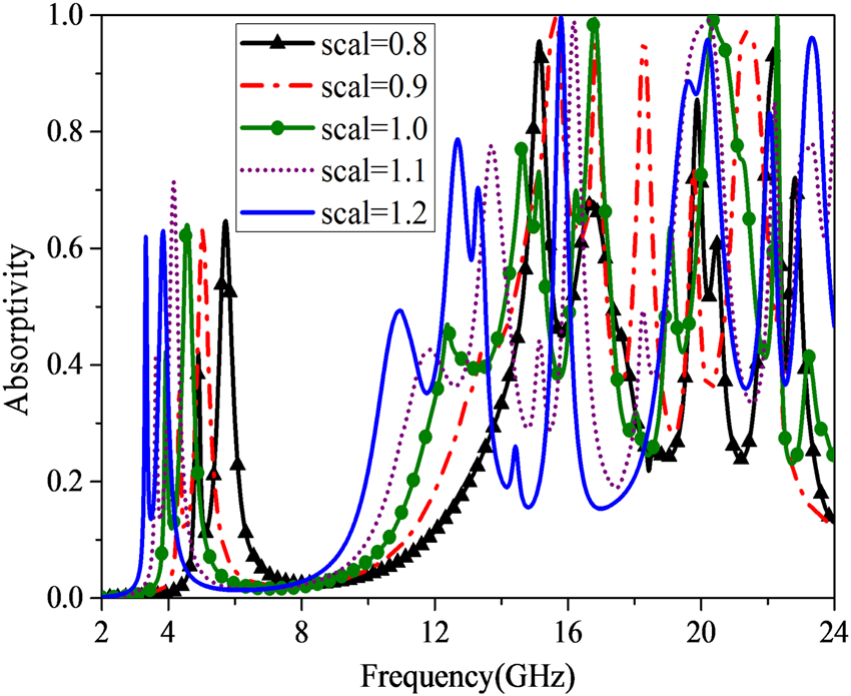
Scalability characteristic of the proposed MMA under normal incidence.

By multiplying the dimensions of top and middle metallic surfaces along x- and y-axis with a constant period S, the scalability characteristic of MMA without resistors is investigated as depicted in Fig. 5. Obviously, the frequency of the absorption peaks located in the relatively low frequency band (specified in 2~6 GHz) decreases with the scaling factor S increases. The corresponding absorptivity of the first absorption peak surges with the scaling factor reaching up to 1.2, while that of the second absorption peak remains nearly unchanged. Furthermore, there is another new absorption peak arising in 9~14 GHz band as the scaling factor S increases. Whilst the frequency of the new absorption peak decreases with the scaling factor S increases. The corresponding absorption peaks with the scaling factor S of 0.9, 1.0, 1.1, 1.2 are located at 13.4 GHz, 12.38 GHz, 11.84 GHz, 10.96 GHz, respectively.

**Figure 6:**
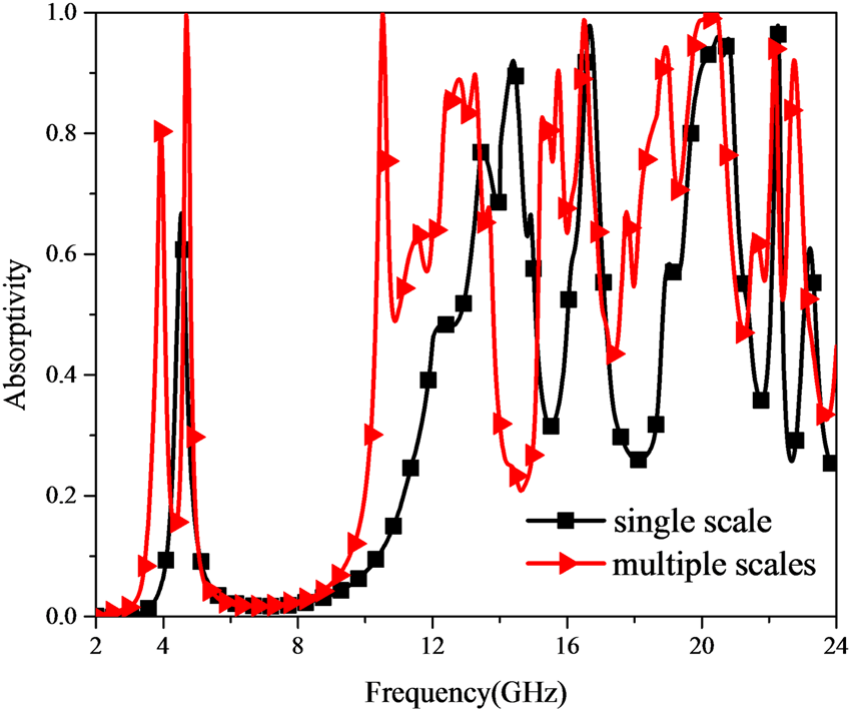
Frequency response of absorption under normal incidence for MMA with single scale and multiple scales.

To be in convenience for following analysis, the basic unit cell to be analyzed is made up of two kind diagonally-arranged sub-cells with scaling factor of 1.0 and 1.2. The absorption property of the MMA under normal incidence has been exploited, while that of the MMA with single scale S = 1.0 has also been illustrated for comparison, as shown in Fig. 6. In the relative low frequency band, there are two sharp splitting absorption peaks at 3.94 GHz and 4.68 GHz with absorptivity of 0.806 and 0.996 for multi-scale MMA, while there is only one single absorption peak valuing 0.668 at 4.54 GHz for single-scale MMA. The absorption difference mentioned above could boil down to the part with S = 1.2. Different lengths of the current-path lead to different resonances. Meanwhile, in the relative high frequency band, the absorption frequency shifts towards lower frequency to some extent. And three broadband absorption humps appear around 11.9 GHz, 16 GHz and 19.3 GHz for multi-scale MMA as revealed in red curve. Further investigation of multiple scalabilities effect on absorbance is detailed in Supplementary Materials.

### Loading of lumped resistors effect

**Figure 7:**
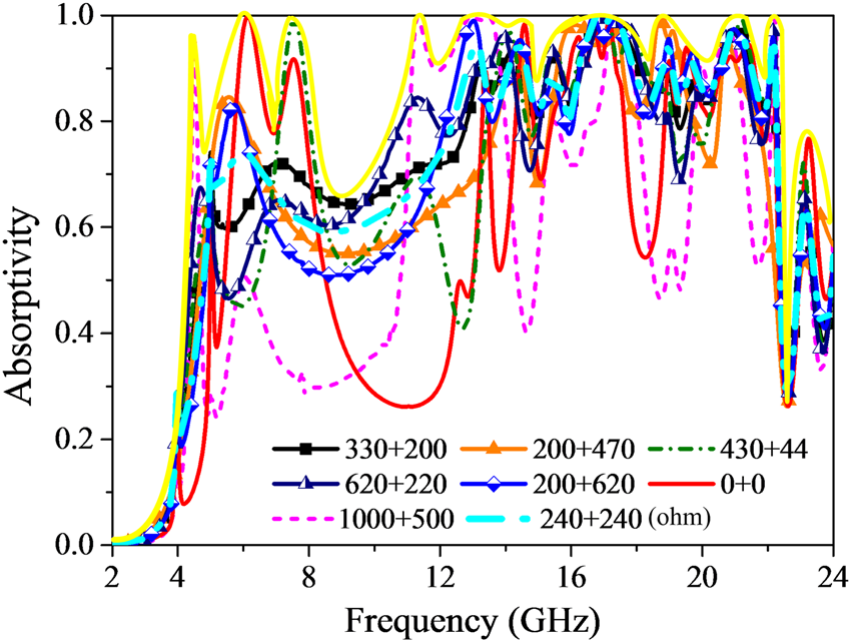
Absorption response versus frequency loaded with different resistor values.

The basic unit cell of finally designed MMA consists of two sub-cells with scaling factor of 0.8 and 1.0, respectively. To further improve the absorbance performance and be easy for fabrication, four lumped resistors are individually added to S = 0.8 and S = 1.0 parts only on the top metallic surface with values of R1 and R2, as depicted in dark grey parts in Fig. 1. The influence of lumped resistors values on absorption property is illustrated in Fig. 7. As clearly plotted in bright yellow solid line, once the mounted resistors values can be tuned arbitrarily, the absorption performance could nearly cover the range from 4 GHz to 24 GHz with high absorption efficiency. It is worth pointing out that by synthesizing the aforementioned three factors, we can achieve the desired absorption performance with high efficiency more easily.

**Figure 8:**
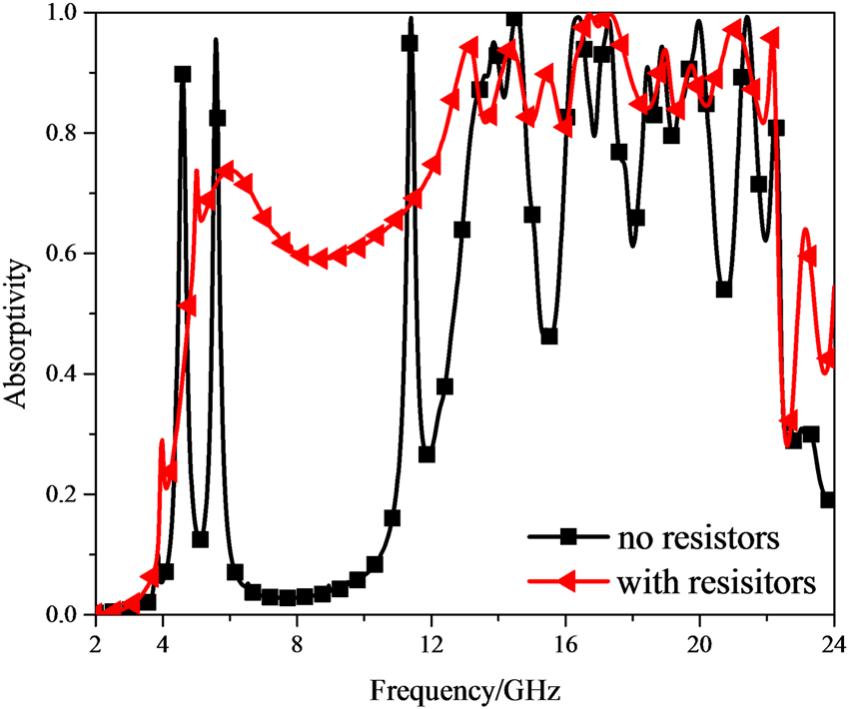
Absorption response versus frequency under normal incidence for MMA with and without resistors.

**Figure 9:**
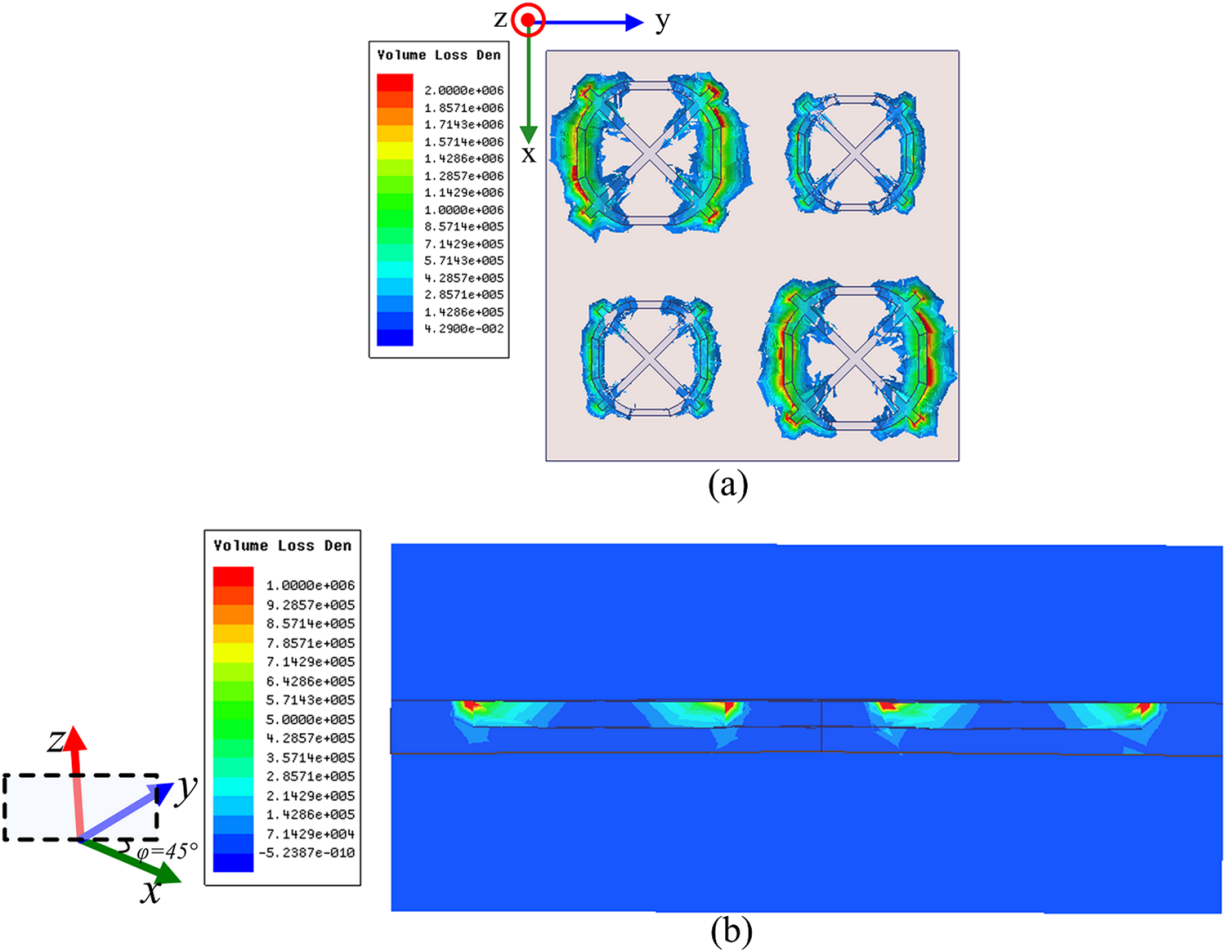
Dielectric (volume) loss in double-layered resistor-loaded MMA at 5.6 GHz. (**a**) Top view and (**b**) transverse view at *φ* = 45° plane.

To balance the absorption performance in low and high frequency band and be easy of fabrication, all lumped resistors in the finally designed MMA are with a value of 240 ohm (water-blue line in Fig. 7). As shown in Fig. 8, in contrast with the case without resistors, when lumped resistors mounted on the SSR, there is a dramatically increase in the absorptivity from 4 GHz to 24 GHz, especially around 8 GHz, 12 GHz, 15.5 GHz and 20.8 GHz. Meanwhile, it is worth noting that a gentle slope belt appears in the absorption band ranging from 5 GHz to 10 GHz instead of two sharp absorption peaks, as depicted in the red curve. To get an insight into the mechanism of absorption difference, the volume loss at 5.6 GHz is shown in Fig. 9. The dielectric losses occur mainly in between the top and middle metallic layers of S = 1.0 part, especially strongly close to the top surface. This is attributed to the lumped resistors loaded on the top metallic layer. Consequently, the modest Fabry-Perot interference and the Ohmic loss in lumped resistors contribute to the gentle absorption belt from 5 GHz to 10 GHz. The induced surface current distributions at 5.6, 6.6 and 15.5 GHz are attached in Supplementary Materials to further exploit lumped resistors effect on absorbance.

### Polarization insensitive and wide incident angle absorption

**Figure 10:**
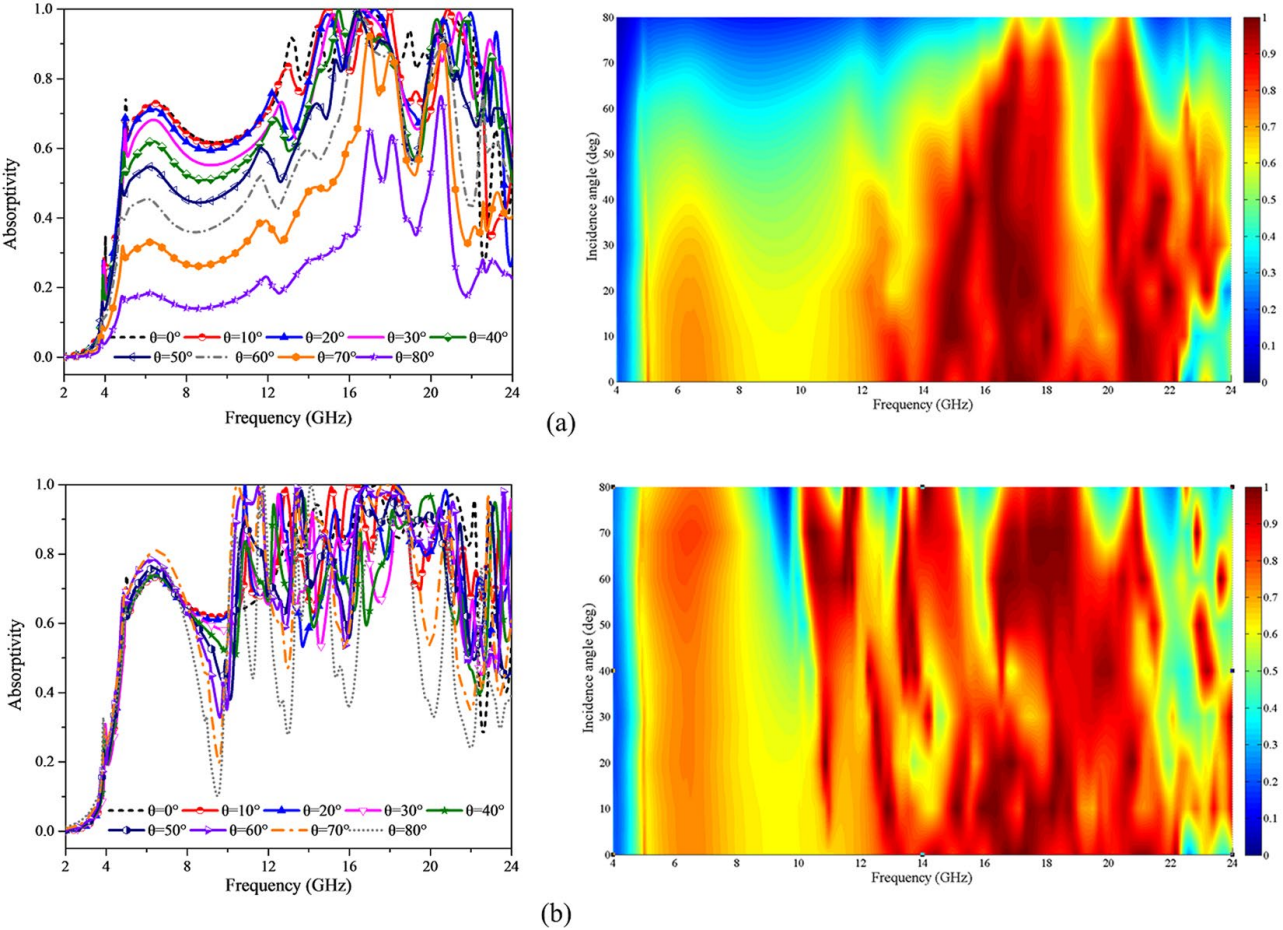
Simulated absorption response and absorption spectra under oblique incidence for (**a**) TE and (**b**) TM polarizations.

**Figure 11:**
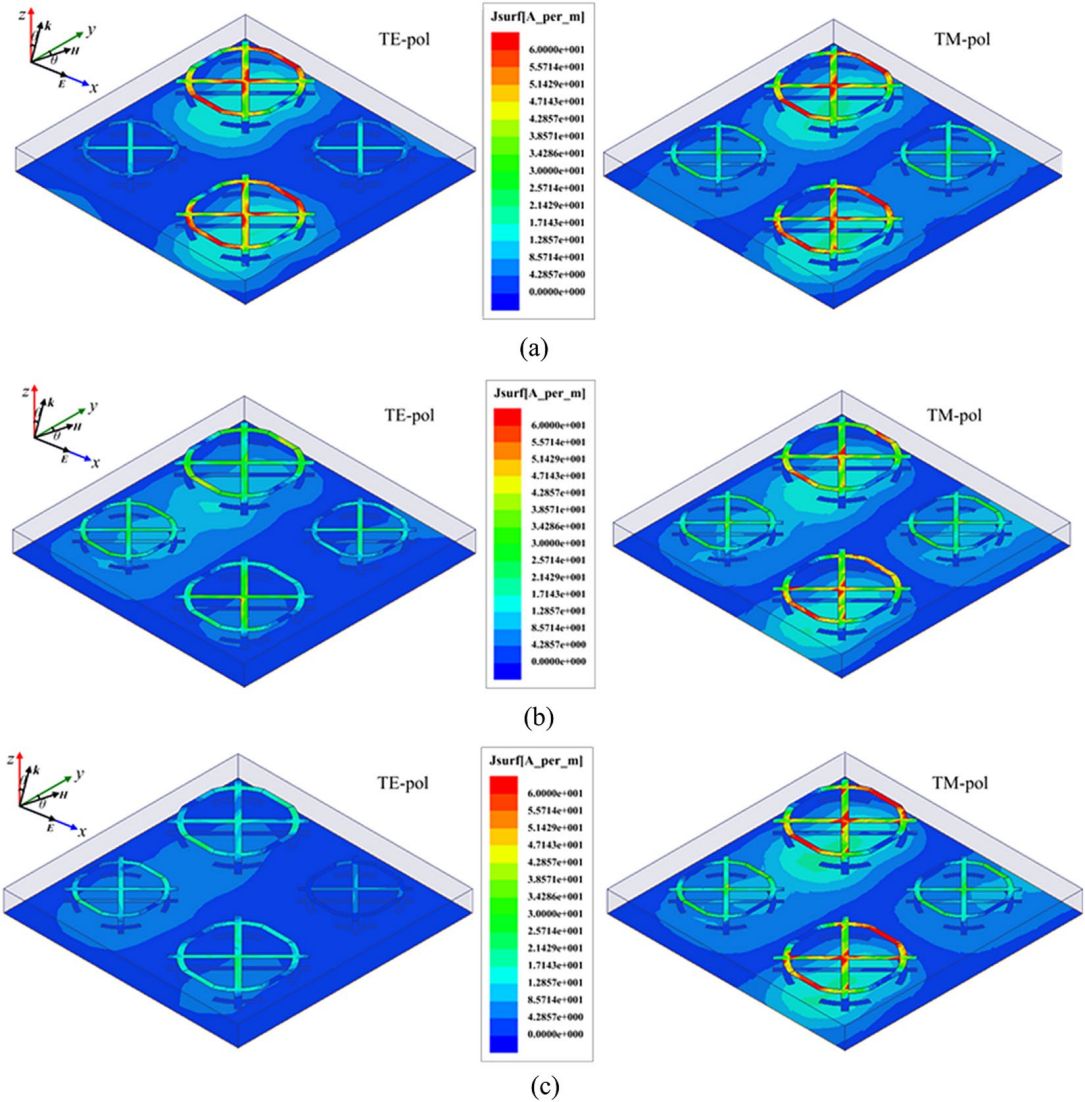
Surface current distribution excited at 6.5 GHz for oblique incident angle *θ* of (**a**) 0 deg, (**b**) 40 deg, (**c**) 80 deg.

In practical applications, the incident EM wave usually impinges on an object target from arbitrary directions. Hence, the absorption performance of the finally proposed MMA under oblique incident wave for transverse electric (TE) and transverse magnetic (TM) polarizations has also been taken into consideration, as depicted in Fig. 10. The proposed MMA demonstrates wide incident angle insensitivity for both TE and TM polarizations. For TE polarization, there exhibits satisfactory absorption performance all along as the incident angle increases from 0° to 50°. When the incident angle reaches up to 60° the absorption performance degrades sharply. Nevertheless, it is worth noting that the absorption performance in the vicinity of 16.9 GHz, 18 GHz and 20.5 GHz keeps remaining over 0.9 as the incidence angle varies from 0° to 70°. As for the case with TM-polarized incidence, the absorption performance in the relatively low frequency band hold steadily as the incidence angle θ increases from 0° to 80°. In contrast, there are some bigger fluctuations in the relatively high frequency band with the increase of incident angle. However, there still depicts a satisfactory absorption performance as the incident angle increases up to 70° over the whole frequency range. When the incidence angle reaches 80°, the absorption performance around 11.76 GHz, 14.7 GHz and 18.56 GHz still remains over 0.95. As a whole, compared with TE polarization, the absorption performance against incident angle degrades unobviously for TM polarization. To exploit the absorption difference between TE and TM polarization, the induced surface currents at 6.5 GHz for oblique incidence are demonstrated, as shown in Fig. 11. For the case of 6.5 GHz, the induced surface current fades with the increase of incident angle for TE polarization, while it holds nearly unchanged until the incidence angle reaching up to 80° for TM polarization. It is worth mentioning that all comparisons are made under the same quantity.

To get an intuitive insight into the absorption performance, a color plot involved absorption performance with respect to both frequencies and incident angles is introduced. It is intuitively evident that the absorption performance for TM polarization exhibits a better performance of incident angle stability in comparison with the case of TE polarization.

**Figure 12:**
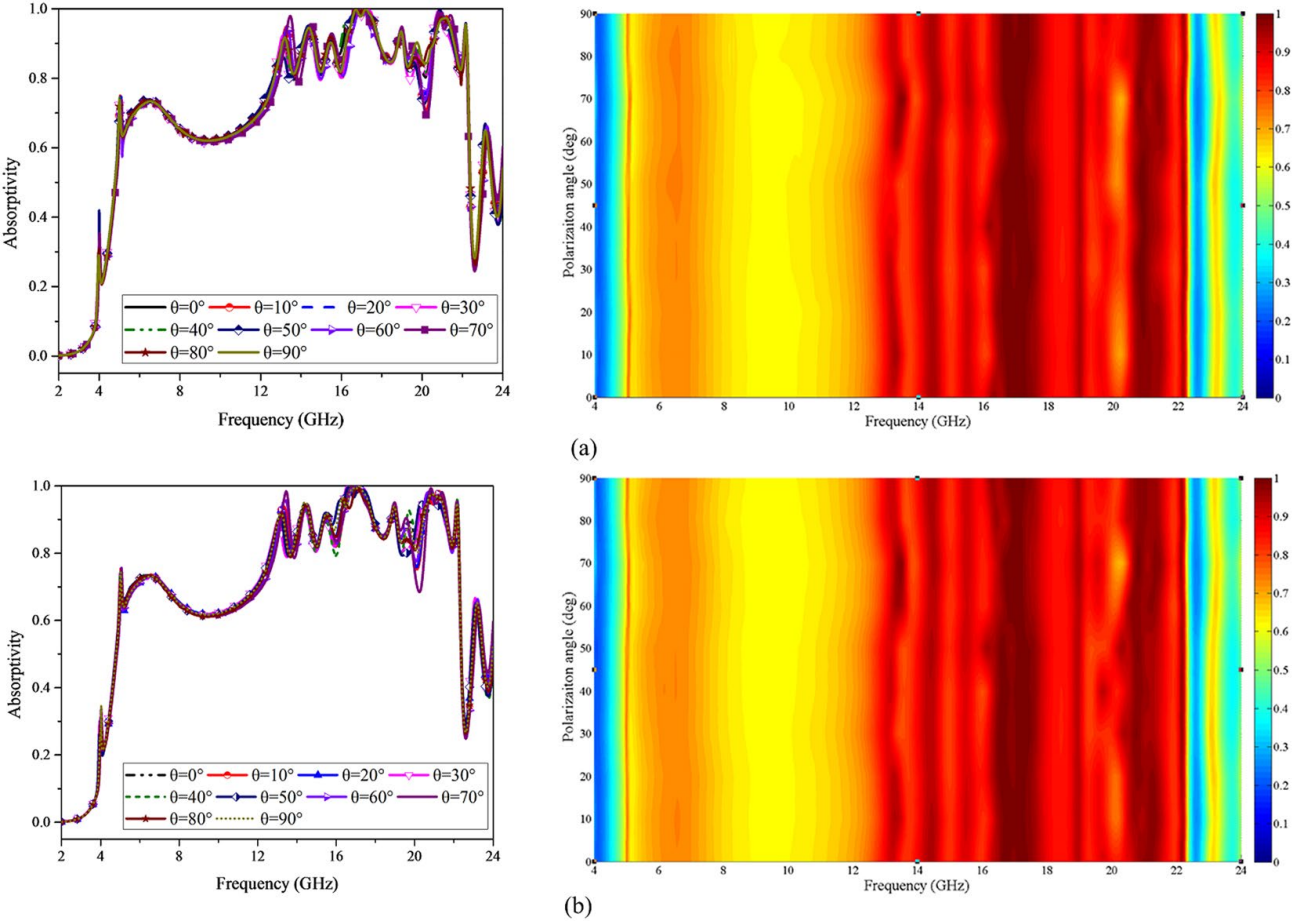
Simulated absorption response and absorption spectra for different polarization angles for (**a**) TE and (**b**) TM polarizations.

Besides, the proposed MMA presents wide polarization angle insensitivity with tiny changes as the polarization angle varies from 0° to 90° for both TE and TM polarizations, as illustrated in Fig. 12. A color plot involving the absorption performance against polarization angles and frequencies for both TE and TM polarizations has also been given. As can be visually observed, the absorption performance remains nearly unchanged with the change of polarization angles. The phenomenon mentioned above can be ascribed to the four-fold symmetry along the x- and y-axis.

## Experimental Results

**Figure 13:**
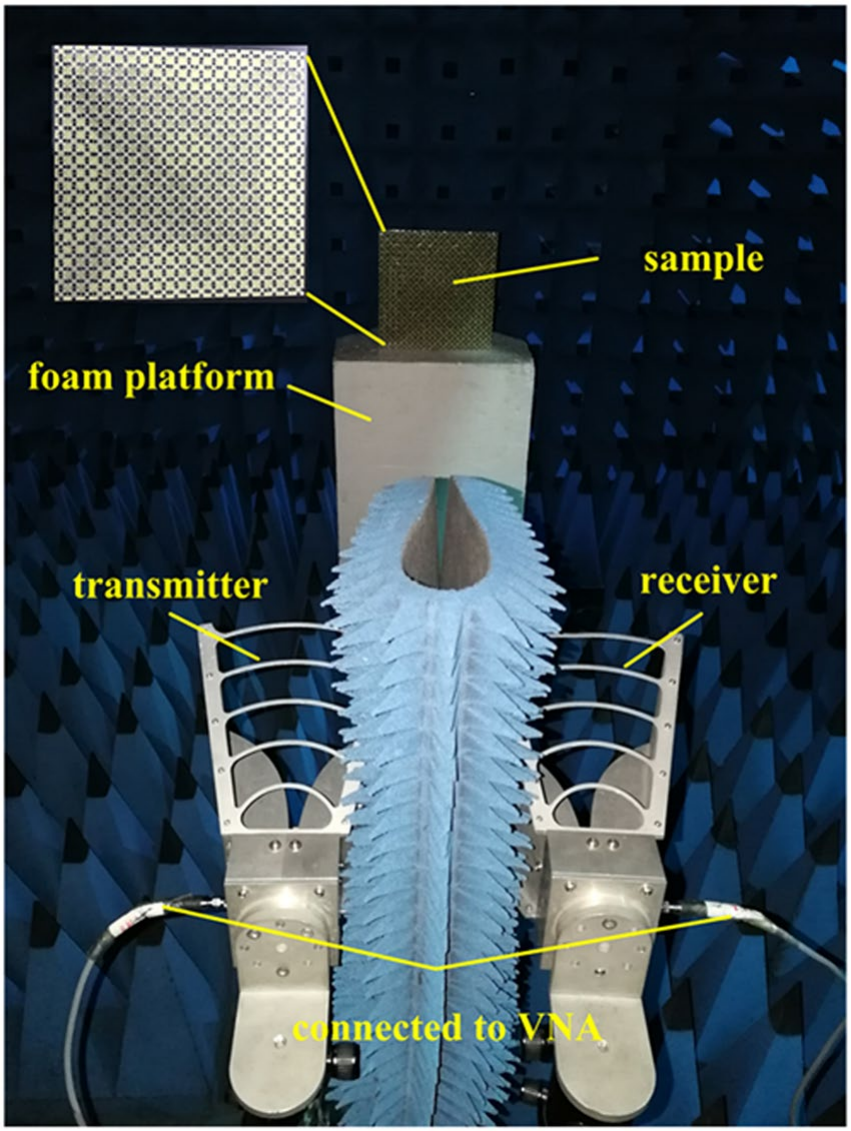
Photograph of the fabricated prototype, measurement circumstance and experimental setup.

**Figure 14:**
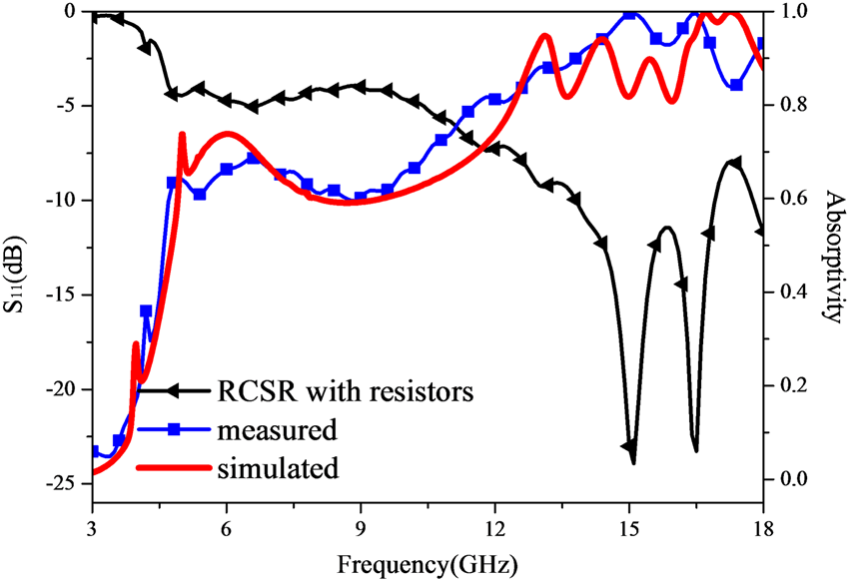
The measured reflectivity and absorptivity response of the fabricated prototype along with the simulated one.

In order to validate the simulated results and further verify the ultra-wideband absorption property, the prototype was fabricated and measured for normal incidence in a microwave anechoic chamber, as exhibited in Fig. 13. The scattering performance of the fabricated sample is evaluated by the transmission coefficients obtained from vector network analyzer. Restricted by the experimental equipment, only reflected power ranging from 2 GHz to 18 GHz has been measured. Before testing the fabricated sample, the reflected power from a same-sized metallic board was measured for reference. The difference between the reflected power from the fabricated sample and that of metallic board determines the actual reflection. Thereafter, the absorptivity is calculated from the measured actual reflection based on the equation mentioned in Sect. 2. The comparison between measured and simulated absorptivity under normal incidence is demonstrated in Fig. 14. As clearly depicted the measured reflectivity over −5 dB nearly covers the range from 6 GHz to 18 GHz (nearly 100% bandwidth), over −8 dB band from 12.6 GHz to 18 GHz (35.3% bandwidth). On the whole, a good agreement has been observed between measurement and simulation. However, some discrepancies between theoretical and experimental results still exist. The size of the fabricated MMA is fixed in practice while being infinite periodic structure in simulation. The fabrication tolerance of the metallic patterns on the top surface, the mounted lumped resistors and the combination of two dielectric layers are important factors that affect experimental precision. All in all, the consistency between simulated and measured results verifies the proposed design capable of ultra-wideband absorptivity.

## Discussion

An ultra-wideband, polarization-independent and wide incident angle MMA has been proposed. Multiple scalability, multiple metallic layers together with lumped resistors are employed together to broaden the absorption bandwidth based on the synergetic effects of Fabry-Perot interference and Ohmic loss. The overall thickness of the designed absorber is only 2.4 mm, thus making the whole structure compact. An ultra-wideband absorbance is achieved nearly in 4~22 GHz, with two gentle slope belts absorptivity over 60% and 80% in 4~12 GHz and 12~22 GHz, respectively. The four-fold symmetric structure makes the finally proposed MMA insensitive to polarization and incident angles. Simulated and measured results have verified the finally proposed MMA capable of ultra-wideband absorbance, which provides a good candidate for EM stealth application.

## Methods

### Numerical simulations

Numerical simulations were carried out using commercial simulation software Ansys HFSS to investigate the EM properties of the MMA. Floquet port excitations and master/slave boundaries are performed to simulate the infinite 2-D periodic boundary condition. When it comes to parameters optimization, control variate method i.e. only one factor is different at a time is adopted to clearly identify the effect of that single factor.

### Sample fabrications and measurements

The metallic structures are built on two 1.2-mm-thick FR4 dielectric boards measuring 280 × 280 mm^2^ through standard printed circuit board technology, whilst one dielectric layer is mounted with lumped resistors among the metallic SSR gaps on the top surface by hand soldering, while the other is backed with a copper lamination on the other side. These two dielectric layers were glued together to perform the ultra-wideband MMA. In order to validate ultra-wideband absorptivity property of the proposed device under normal incidence, backscattering measurement is conducted in an anechoic chamber to minimize the interference from outer environment. The fabricated device is placed vertically on the center of a foam platform, while two identical linear-polarized pyramidal horn antennas working at 1–18 GHz were placed adjacently as transmitter and receiver, respectively. The centers of the device and two horns are in the same height and the distance between them is 1.8 m, which is far enough to satisfy the far-field test condition and guarantee the incidence illuminating on the device be treated as plane wave. Gate-reflect-line calibration in time-domain analysis kit of vector network analyzer (VNA) has been employed to further eliminate undesirable signals in the environment. The two horn antennas for transmitting and receiving are connected to the two ports of the vector network analyzer Agilent N5230C to evaluate reflected power on transmission coefficients of S_21_.

## Electronic supplementary material

Supplementary Information
