# Supplementary material for: Angular- and Polarization-insensitive Ultrathin Double-layered Metamaterial Absorber for Ultra-wideband Application
Source: Sci Rep. 2018 Jun 25;8:9627. doi: 10.1038/s41598-018-28041-5 (PMC6018427; doi:10.1038/s41598-018-28041-5)
Supplement: Supplementary file 1 — Supplementary Information [file 41598_2018_28041_MOESM1_ESM.doc]

Supplementary Materials for

**Angular- and Polarization-insensitive Ultrathin Double-layered Metamaterial Absorber for Ultra-wideband Application**

Li Li Cong^1, *^, Xiang Yu Cao^1, *^, Tao Song^2, +^, Jun Gao^1, +^, Jun Xiang Lan^1, +^

^1^Information and Navigation College, Air Force Engineering University, Xi’an 710077, China

^2^ Air Defense and Missile College, Air Force Engineering University, Xi’an 710077, China

^*^ Correspondence and requests for materials should be addressed to L. L. C. (email:1183068955@qq.com) or X. Y. C. (gjgj9694@163.com)

^+^ These authors contributed equally to this work.

To further exploit the mechanism of three factors effect on the absorption performance, the current distributions are demonstrated below to better interpret the ultra-wideband absorbance.

- **Multiple metallic layers effect**

To further investigate metallic layer effect on absorbance, the surface current distributions at 3.82 GHz for MMA with single and double metallic layers are depicted in Fig. 1. Apparently, for single metallic layer, there exhibits nearly no excited surface current at 3.82 GHz which contributes to a close-to-zero absorptivity, while for double metallic layers, modest excited surface current mainly concentrates on the bars of the top surface with little current on the central part of backing plate.


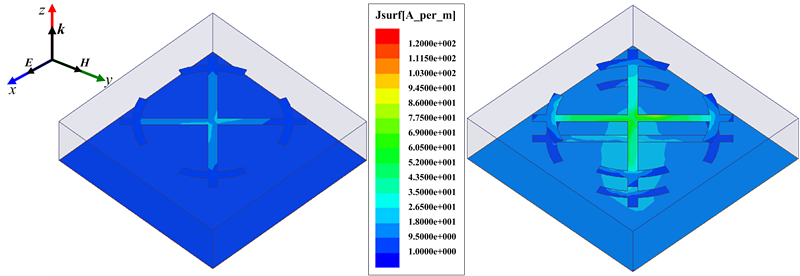


**Figure 1.** Surface current distributions for MMA with single metallic layer and double metallic layers excited by normal incidence at 3.82 GHz.

- **Multiple scalabilities effect**

To further investigate multiple scalabilities effect on absorbance, the surface current distributions at 3.94 GHz and 12.78 GHz are demonstrated in Fig. 2, respectively. For incident EM wave at 3.94 GHz, there exhibits rather little excited surface current for single scale MMA, which results in nearly zero absorptivity. While strong surface current excited on the orthogonal bars of S=1.2 part on the top surface and relative position of backing plate, which contributes to an abrupt absorption peak. Meanwhile, as clearly shown in Fig. 3, the excited currents at 3.94 GHz mainly concentrate on S=1.2 part, while that at 4.68 GHz mainly concentrate on S=1.0 part. The induced currents resonant at different locations indicate that the splitting resonances correspond to different fundamental modes, not to the symmetric and anti-symmetric modes. The lager length of the current-path on S=1.2 part leads to lower resonant frequency, thus resulting in resonance splitting at 3.94 GHz and 4.68 GHz. For 12.78 GHz, the weakly induced surface current mainly distributes on the middle layer and backing plate for single scale MMA, while that mainly concentrates on the SSRs of top and middle metallic layers for multi-scale MMA. It is noted that the strongly induced current mainly concentrates at S=1.0 part with that subordinately at S=1.2 part for multi-scale MMA. Due to the excited current disperses almost evenly for either part of S=1.0 and S=1.2, the multi-scale MMA exhibits a gentle absorption peak at 12.78 GHz. It is worth pointing out that three split absorption peaks occurs at 12.42 GHz, 12.78 GHz and 13.26 GHz. For induced current distributions at 12.42 GHz and 12.78 GHz, we can clearly observe in Fig. 4(a) and (b) that the current mainly induced on the S=1.2 part for 12.42 GHz, while mainly on the S=1.0 part otherwise. From the symmetric current distribution, we can conclude that the reason for resonance splitting is not mode splitting. The different lengths of current-path result in different resonant frequencies. However, when it comes to 13.26 GHz, the induced current distributes mainly on S=1.0 part, and the direction of induced current flow is opposite in comparison with that at 12.78 GHz. Hence, the splitting absorption peaks at 12.78 GHz and 13.26 GHz are attributed to the mode splitting based on the coupled-mode theory.


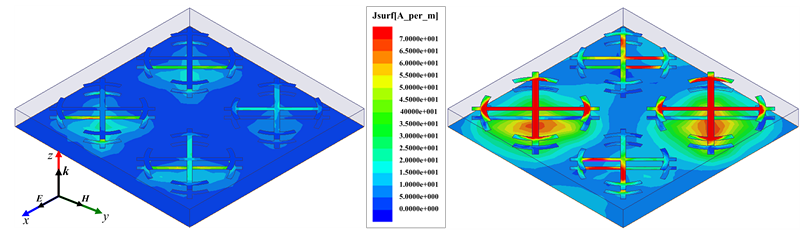


(a)


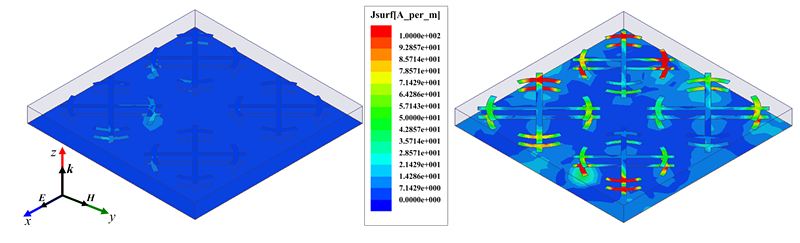


(b)

**Figure 2.** Surface current distributions of the single-scale and multi-scale MMA excited by normal incidence at (a) 3.94 GHz, (b) 12.78 GHz. (Left: single-scale MMA, right: multi-scale MMA)


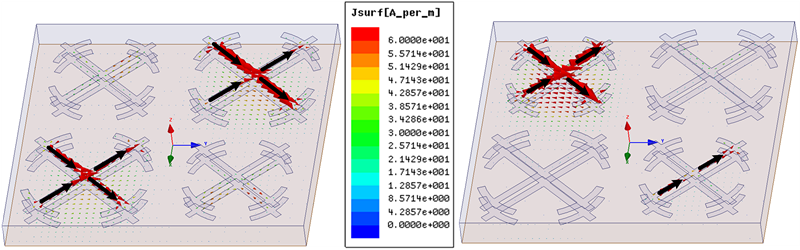


**Figure 3.** Current distributions across metallic surface for multi-scale MMA at 3.94 GHz and 4.68 GHz.


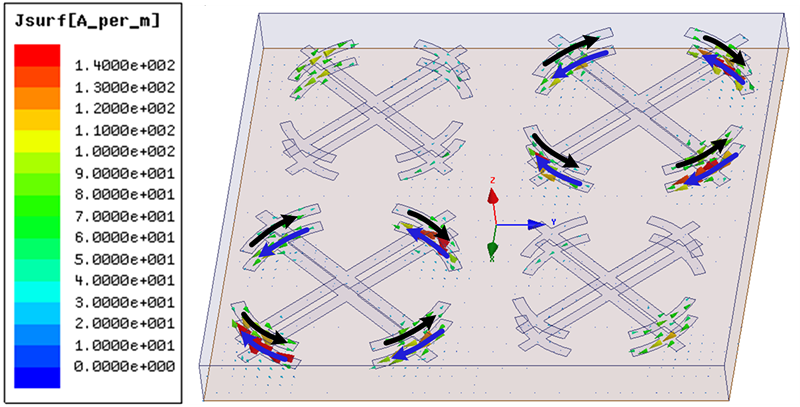


(a)


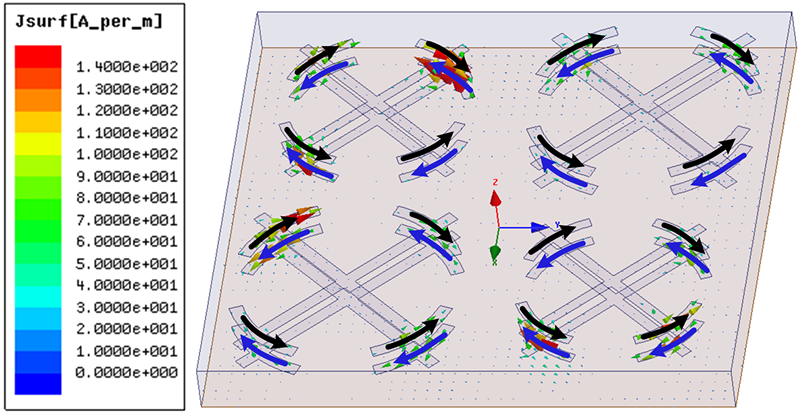


(b)


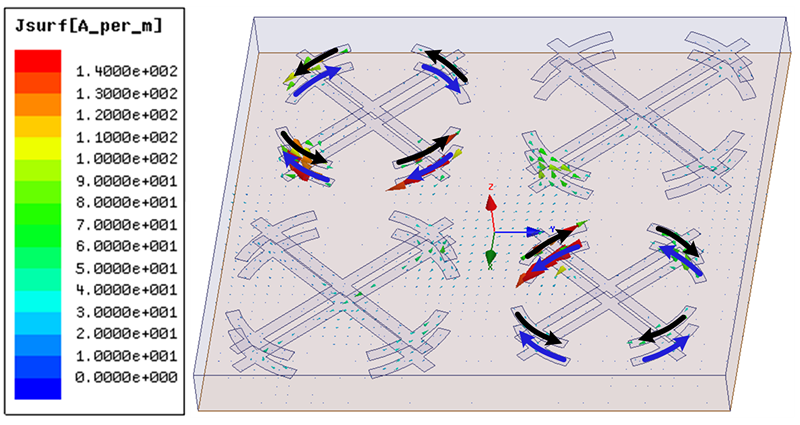


(c)

**Figure 4.** Current distributions across metallic surface for multi-scale MMA at (a) 12.42 GHz (b) 12.78 GHz and (c) 13.26 GHz.

- **Loading of lumped resistors effect**

To further investigate the effect of loading lumped resistors on absorbance, the induced surface current distributions at 5.6 GHz, 6.6 GHz and 15.5 GHz are illustrated in Fig. 5, respectively. For the case of MMA without lumped resistors, the strong absorption peak at 5.6 GHz results from the powerful induced surface current on the orthogonal bars of S=0.8 part on the top metallic layer and relative position on the backing lattice. In contrast, with the resistors loaded, the surface current mainly concentrates on the top metallic layer of S=1 part and relative position of backing metallic plate, together with the gaps where the lumped resistors are loaded. At 6.6 GHz, for the case without resistors, the induced current only appears on the top metallic surface, which cannot form the Fabry-Perot cavity interference. Nevertheless, the case of MMA with lumped resistors at 6.6 GHz is under the same circumstance with that at 5.6 GHz. When it comes to the case of 15.5 GHz, the induced resonant current mainly gathers at the four SSRs of S=1 part on the top metallic surface without resistors, while destructive Fabry-Perot interference occurs between the middle metallic layer of S=1 part and relative position of backing lattice together with the Ohmic loss in lumped resistors.


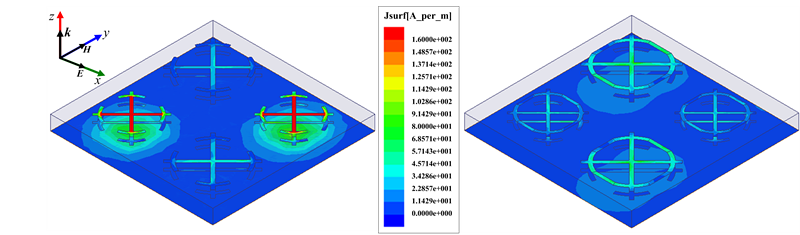


(a)


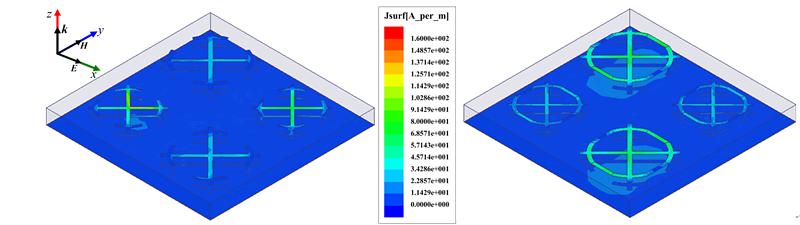


(b)


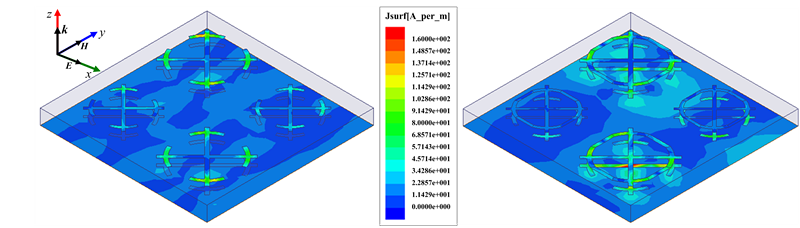


(c)

**Figure 5.** Surface current distributions of the MMA with and without lumped resistors excited by normal incidence at (a) 5.6 GHz, (b) 6.6 GHz, (c) 15.5 GHz. (Left: without resistors, right: with resistors)
